# Co-transplantation with adipose-derived cells to improve parathyroid transplantation in a mice model

**DOI:** 10.1186/s13287-020-01733-4

**Published:** 2020-05-26

**Authors:** Qiuxia Cui, Dan Zhang, Deguang Kong, Jianing Tang, Xing Liao, Qian Yang, Jiangbo Ren, Yan Gong, Gaosong Wu

**Affiliations:** 1 Department of Thyroid and Breast Surgery, Zhongnan Hospital of Wuhan University, 169 Donghu Road, Wuhan, China; 2 Department of Anesthesiology, Xiamen Branch, Zhongshan Hospital, Fudan University, Shanghai, China; 3 Department of Biological Repositories, Zhongnan Hospital of Wuhan University, 169 Donghu Road, Wuhan, China

**Keywords:** Parathyroid transplantation, Angiogenesis, Stromal vascular fraction, Adipose-derived stem cell, Endothelial cell

## Abstract

**Background:**

Accidentally removed parathyroid glands are still challenging in neck surgery, leading to hypoparathyroidism characterized with abnormally low levels of parathyroid hormone. Parathyroid auto-transplantation is usually applied in compensation. To improve the efficiency of parathyroid transplantation, we introduced a method by co-transplanting with adipose-derived cells, including stromal vascular fractions (SVFs) and adipose-derived stem cells (ADSCs), and investigated the underlying molecular mechanisms involved in parathyroid transplantation survival.

**Methods:**

Rat and human parathyroid tissues were transplanted into nude mice as parathyroid transplantation model to examine the effects of SVFs and ADSCs on grafts angiogenesis and survival rates, including blood vessel assembly and parathyroid hormone levels. Several angiogenic factors, such as vascular endothelial growth factor (VEGF)-A and fibroblast growth factor (FGF) 2, were assessed in parathyroid grafts. The effects of hypoxia were investigated on ADSCs. The modulatory roles of the eyes absent homolog 1 (EYA1), which is vital in parathyroid development, was also investigated on angiogenic factor production and secretion by ADSCs. All experimental data were statistically processed. Student’s *t* test was used to assess significant differences between 2 groups. For multiple comparisons with additional interventions, two-way ANOVA followed by Tukey’s post hoc test was performed. *P* < 0.05 was considered as significant.

**Results:**

SVFs improve rat parathyroid transplantation survival and blood vessel assembly, as well as FGF2 and VEGF-A expression levels in parathyroid transplantation mice. Functional human parathyroid grafts have higher microvessel density and increased VEGF-A expression. The supernatant of ADSCs induced tubule formation and migration of human endothelial cells in vitro. Hypoxia had no effect on proliferation and apoptosis of human ADSCs but induced higher angiogenic factor levels of VEGF-A and FGF2, modulated by EYA1, which was confirmed by parathyroid glands transplantation in mice.

**Conclusions:**

Adipose-derived cells, including ADSCs and SVFs, improve parathyroid transplantation survival via promoting angiogenesis through EYA1-regulating angiogenetic factors in vitro and in vivo. Our studies proved an effective method to improve the parathyroid autotransplantation, which is promising for clinical patients with hypoparathyroidism when parathyroid glands were accidentally injured, removed, or devascularized.

## Background

Surgery for thyroid, throat, and other head-neck tumors, as well as parathyroidectomy in patients with parathyroid hyperplasia, usually leads to hypoparathyroidism characterized with abnormally low levels of parathyroid hormone (PTH) [1]. The incidence rate of accidental parathyroidectomy was reported 5.2 to 21.6% in thyroidectomy, especially with central lymph nodes dissection [2, 3]. The incidence of transient hypoparathyroidism was reported ranging from 28.4 to 49%, and that of permanent hypoparathyroidism ranged from 1.7 to 13% [4]. Parathyroid autotransplantation (PA) is an important supplemental strategy to prevent hypoparathyroidism when parathyroid glands were injured, removed, or devascularized inadvertently during surgery. Although the PA procedure and skills have been well developed, the effectiveness of PA was not ideal [5]. Our preliminary clinical researches showed that the grafts with surrounding adipose tissues appeared with lower incidence of permanent hypoparathyroidism than those grafts without adipose tissues, suggesting that adipose tissue might be a factor to improve transplantation survival.

Subcutaneous adipose tissue is readily accessible due to increased obesity and liposuction surgeries [6]. More attention of regenerative medicine has been paid on adipose-derived stromal vascular fractions (SVFs) [7], which are composed of vascular endothelial cells, adipose progenitor cells, stromal cells, and blood cells [8]. Previous studies suggested that SVFs were osteogenic, chondrogenic, adipogenic, and angiogenic [9]. In addition, SVFs were also reported to promote blood vessel growth during wound healing [10]. Other studies indicated that adipose-derived stem cells (ADSCs) promoted angiogenesis via inducing vascular endothelial growth factor (VEGF) through autocrine and paracrine pathways [11]. VEGF-A and fibroblast growth factor (FGF) 2 were reported to modulate angiogenesis of transplanted parathyroid glands [12]. However, the studies on the effects of adipose-derived cells or extracellular vesicles on parathyroid grafting are still limited. Whether ADSC will play a role parathyroid glands transplantation is a major question to be solved in this study. Therefore, it is interesting and important to investigate the roles of adipose tissues in PA efficiency and get more comprehensive understanding of the interaction between adipose tissues and parathyroid grafts.

Besides, hypoxia was reported to benefit ADSC functions including proliferation, differentiation, and growth factor secretions [13, 14]. Under hypoxia, hypoxia-inducible factor (HIF)-1α translocated into the nucleus and regulated target gene expression [15]. HIF-1α was reported inducing angiogenesis through VEGF-A [16]. HIF-1α was also reported to affect mesenchymal stem cell (MSC) behaviors including cell viability, proliferation, differentiation, and migration [17]. In addition, fat grafting with MSCs showed their regenerative and angiogenic potentials [18]. However, the effects of hypoxia on ADSCs remain unknown [19]. Therefore, detailed investigation on hypoxic ADSCs in parathyroid gland grafting is necessary.

The eyes absent homolog 1 (EYA1) is a transcription factor involved in cell proliferation, tissue differentiation, and organ development during embryonic development, especially necessary in parathyroid development. Deficiency of EYA1 resulted in abnormal parathyroid glands [20]. As a component of DACH1/SIX1/EYA1 complex, EYA1 is phosphorylated and coactivated with SIX1 in the nucleus, and the complex interacts with TGFβ/Smads signaling pathway during differentiation [21]. EYA1 promotes cell proliferation and inhibits apoptosis and DACH1 participates in angiogenesis procedure by inhibiting the expression of FGF2 [22]. A schematic model shows how SIX1/EYA1 coordinates to control both mesenchymal development and epithelial branching in the lung [23]. It was reported that higher EYA1 expression was associated with the increase of VEGF-A [24].

The in vitro and in vivo experiments found that EYA1-expressing tumor cells promoted HUVEC migration and tube formation by regulating VEGF-A, but the expression of VEGF-A was inhibited when eliminating EYA1 [25]. In addition, EYA family plays a key role in regulating cell behavior and is related with some organ development relative vascular diseases [26, 27]. VEGF-A is significant in the procedure of angiogenesis [28], which is regulated by the oxygen-sensitive transcriptional activator HIF-1α in response to hypoxic conditions [29, 30]. A recent research discovered that EYA1 induced the expression of VEGF-A with the coordination of HIF-1α, promoting angiogenesis in colorectal tumors [31]. And 6-hydroxybenzylbromarone was found inhibiting the angiogenic procedure by blocking the activation of EYA [32].

Our hypothesis is that ADSCs, existing in the SVFs, improve PA survival via inducing angiogenesis through activating EYA1. We evaluated the effects of ADSCs on angiogenesis in endothelial cell culture in vitro and PA mouse model in vivo. Our results indicated that ADSCs in the SVFs increased PA survival via promoting angiogenesis through DACH1/SIX1/EYA1 pathway, suggesting the beneficial roles of adipose tissues during parathyroid transplantation.

## Methods

### Mouse model of parathyroid transplantation

Male nude mice (BALB/C, 6–8 weeks, 20 ± 5 g body weight) were obtained from Beijing Vital River Laboratory Animal Technology Co., Ltd. (China). These nude mice were anesthetized with inhaled isoflurane gas machine (Shanghai Yuyan Scientific Instrument Co. Ltd., China). A subcutaneous pocket was made on the back or at the right side of the groin, and 1 rat parathyroid gland or 3–5 pieces of minced human parathyroid tissues were transplanted into the subcutaneous pocket. For SVF experiments, a mixture of SVFs (20 μl, 5 × 10^4^ cells/μl) was simultaneously implanted for the experimental group, while 20 μl PBS was co-transplanted for the control group. For ADSC experiments, ADSCs (20 μl, 5 × 10^3^ cells/μl) with or without EYA1 deficiency were simultaneously implanted. The pocket was closed with nonabsorbent monofilament for identification of the implantation site. All the animal experiments were approved by the Animal Center of Tongji Medical College, Huazhong University of Science and Technology, and Wuhan University, in accordance with ARRIVE guidelines, and performed in accordance with relevant guidelines including US National Research Council’s Guide for the Care and Use of Laboratory Animals.

### Plasma levels of intact parathyroid hormone

Blood was collected from the retro-orbital plexus of mice under anesthesia before transplantation, and on the 5th, 12th, and 19th day after transplantation. The plasma levels of PTH, as well as VEGF-A, IFN-β, and TGF-β, were measured by species-specific enzyme-linked immunosorbent assays (ELISA) with Rat ELISA Kit (Elabscience, Wuhan, China) according to the manufacturer’s instructions.

### Human parathyroid tissues

Fresh parathyroid tissues were obtained from 2 patients with parathyroidectomy for secondary hyperparathyroidism after their consent. The normal tissues were identified by H&E staining and collected for parathyroid transplantation, and they were cut into small fragments sized of 2 × 2 × 1 mm^3^. The boiled tissues were inactivated by heating in a water bath at 56 °C for 30 min as the control group. This study was approved by the Ethics Committee for Human and Animal Research of Tongji Hospital, Tongji Medical College of Huazhong University of Science and Technology, and Wuhan University. Free prior written informed consents were obtained from the 2 donors. All experiments were carried out in accordance with the Declaration of Helsinki and other recognized standards.

### Rat parathyroid tissues

The parathyroid glands of 28 Sprague-Dawley (SD) male rats, (~ 8 weeks of age, 260–350 g body weight) were identified by 5-aminolevulinic acid hydrochloride (5-ALA) photosensitization. In brief, 5-ALA powder (Sigma-Aldrich, USA) was suspended in a 0.5 ml of 0.9% sodium chloride solution and intraperitoneally injected (500 mg/kg) into the rats. Animals were anesthetized by inhaled isoflurane gas machine 2 h later, and a vertical skin incision was made at the midline of the neck. Muscle was dissected to the trachea and thyroid gland, and the parathyroid glands were visualized under the illumination of xenon light source (380–440 nm) and an ultraviolet filter to detect fluorescence at the 635 nm wavelength. The pair of parathyroid glands exhibited red fluorescence anterolateral to the thyroid gland. Bilateral parathyroidectomy was performed, and the rats were sacrificed with 4% pentobarbital. All the experiments were approved by the Ethics Committee for Animal Experiments of Tongji Medical College and Wuhan University.

### SVF isolation and ADSC cultivation from human adipose tissues

SVFs were isolated as previously described [33, 34]. Briefly, adipose tissues were obtained from 2 healthy female donors, aged 18 to 30 years, undergoing an abdominal liposuction bariatric procedure with written informed consent. These lipoaspirates were washed with phosphate buffered saline (PBS), finely minced, and enzymatically digested with 2 mg/mL type I collagenase at 37 °C for 40 min. The tissues were then centrifuged to remove buoyant adipocytes, and the top layers were retrieved to obtain the SVFs. Nucleated SVFs (1 × 10^7^ cells) were plated in 10-cm culture plates and cultured in Dulbecco’s modified Eagle’s Medium (DMEM) F12 supplemented with 10% FBS and 1% antibiotic/antimycotic. ADSCs were obtained after 3–4 passages of SVFs cultured in the corresponding medium as reports [8].

### SVF isolation from rat adipose tissues

Adipose tissues were collected from the abdomen area of SD male rats. These lipoaspirates were washed with PBS, finely minced, and enzymatically digested with 2 mg/mL type I collagenase at 37 °C for 40 min. The tissues were then centrifuged to remove buoyant adipocytes, and the top layers were washed with PBS. The resuspended solution was filtered through 70 μm filter twice and centrifuged at 2000 rpm for 10 min. The pellets were washed with PBS for 3 times until the solution is pure clear [35].

### Flow cytometry analysis

The harvested cells were incubated with 10% goat serum (BOSTER Biotechnology, Wuhan, China) at 37 °C for 30 min and washed with ice-cold PBS twice. After incubated with primary antibodies against CD73 (R&D, USA), CD90 (R&D, USA), CD45 (Santa Cruz, USA), and CD31 (Santa Cruz, USA) at 4 °C for 30 min, the cells were stained with FITC-conjugated secondary antibody (Abcam, USA) according to manufacturer’s instructions. After washing with PBS, cells (1 × 10^5^) were acquired in a BD FACSCalibur (BD, Belgium) and analyzed using the Cyflogic version 1.2.1 software (CyFlo, Finland).

### Adipogenic and osteogenic differentiation

For adipogenesis, the ADSCs (90% confluence) were cultured with adipogenic medium composed of DMEM/F12 supplemented with FBS (GIBCO™, USA, 0.1 mL/mL), streptomycin (100 μg/mL), penicillin (100 U/mL), L-glutamine (2 mmol/L), insulin (10 μmol/L), isobutylmethylxanthine (0.5 mmol/L), dexamethasone (1.0 μmol/L), pioglitazone (10 μmol/L), rosiglitazone (0.5 μmol/L), biotin (33 μmol/L), and pathenonate (17 μmol/L) for 3 days. The cells were then cultured for another 3 days with adipogenic medium without isobutylmethylxanthine, biotin, or pathenonate. The presence of intracellular lipid deposits was confirmed with Oil Red O (0.3%, Solarbio, Beijing, China).

For osteogenesis, the ADSCs (90% confluence) were cultured with osteogenic medium (DMEM with 0.1 mM dexamethasone, 0.05 mM ascorbic acid, and 10 mM sodium glycerophosphate). The accumulation of calcium deposits was visualized with Alizarin Red S dye.

### Cell proliferation assay

Cell proliferation was assessed by Cell Counting Kit-8 assay (CCK-8, Sungene Biotech, Tianjin, China). Briefly, cells (5 × 10^3^ cells/well) were seeded in 96-well plates with supernatant of hypoxia- or normoxia-conditioned ADSCs. The CCK-8 reagents were added into each well at a final concentration of 10% 2 days later, and the cells were maintained at 37 °C for 1 h. The absorbance was measured at 450 nm using a Microplate Reader (Bio-Rad, Hercules, CA, USA).

### Wound healing assay

When full confluence, a scratch was with a sterile pipette tip. The cells were washed with PBS and cultured with fresh serum-free culture medium containing hypoxia- or normoxia-conditioned supernatant of ADSCs. Images were taken at 0, 12, and 24 h after scratch, and the areas of wound healing were measured using ImageJ software (NIH, Bethesda, MD, USA).

### Tube formation assay

The 24-well plates (Corning, NY) were coated with 50 μl of growth factor-reduced Matrigel and incubated at 37 °C for 30 min. The human umbilical vein endothelial cells (HUVECs, CL-0122, Procell Life Science & Technology Co., Ltd., Wuhan, China, 2 × 10^5^ cells/well) were seeded into the plates and cultured with ADSC-conditioned medium for 24 h. The tubular formation was analyzed with Angiogenesis Analyzer for ImageJ.

### Immunoblotting

The total protein was extracted with RIPA buffer on ice and then fractionated on 10% sodium dodecyl sulfate-polyacrylamide gels. After being transferred to polyvinylidene fluoride membranes, the proteins were blocked with 5% nonfat dry milk at room temperature for 1 h. After incubated with primary antibodies against mouse VEGF-A, FGF2, HIF-1α, EGF, DACH1 (1:1000 dilution, Santa Cruz, USA), EYA1, SOD2, Smad4, Smad3, AR, TGF-β (1:1000 dilution, Proteintech, China), or SIX1 and vinculin (1:1000 dilution, Sigma- Aldrich, USA) at 4 °C overnight, the membranes were subsequently incubated with horseradish peroxidase-conjugated anti-mouse secondary antibody (1:5000 dilution, Cell Signaling Technology, USA, at room temperature) for 1 h. Protein bands were visualized by chemiluminescence (Bio-Rad Laboratories, Inc., USA), and their intensities were quantified using ImageJ software (National Institutes of Health). Adobe Photoshop CC 2015 (Adobe, California, USA) was applied to analyze the corresponding grayscale.

### Transfection and PCR

The ADSCs were transfected with EYA1 siRNA (sense: 5′- CAGGAAAUAAUUCACUCACAAdTdT-3′; antisense: 5′- UUGUGAGUGAAUUAUUUCCUGdTdT- 3′, Genechem, Shanghai, China) using Lipofectamine 2000 reagent (Genechem) in serum-free DMEM/F12 medium according to the manufacturer’s instructions. The total RNA was extracted from cells using HiPure Total RNA Mini Kit (Magen, Guangzhou, China). RNA concentration was measured with an ultraviolet spectrophotometer. After reverse transcription, the cDNA was used for qPCR. The primers are listed as follows: EYA1 (forward: 5′-GCAGTAGTTTCAGCCCACGA-3′, reverse: 5′-CACCATATGAGGAAATGCCG-3′) and β-actin (forward: 5′-ATGTACGTTGCTATCCAGGC-3′, reverse: 5′-TCTTAATGTCACGCACGAT-3′). The relative expression levels of genes were normalized to β-actin.

### Statistical analysis

All experimental data were statistically processed using SPSS 20.0, ImageJ and Graphpad Prism 7. The data were presented as the mean ± SD. Student’s *t* test was used to assess significant differences between 2 groups. For multiple comparisons with additional interventions, two-way ANOVA followed by Tukey’s post hoc test was performed. *P* < 0.05 was considered as significant.

## Results

### SVFs promote the graft survival of rat parathyroid tissues and vascular assembly of the transplanted parathyroid

**Fig. 1:**
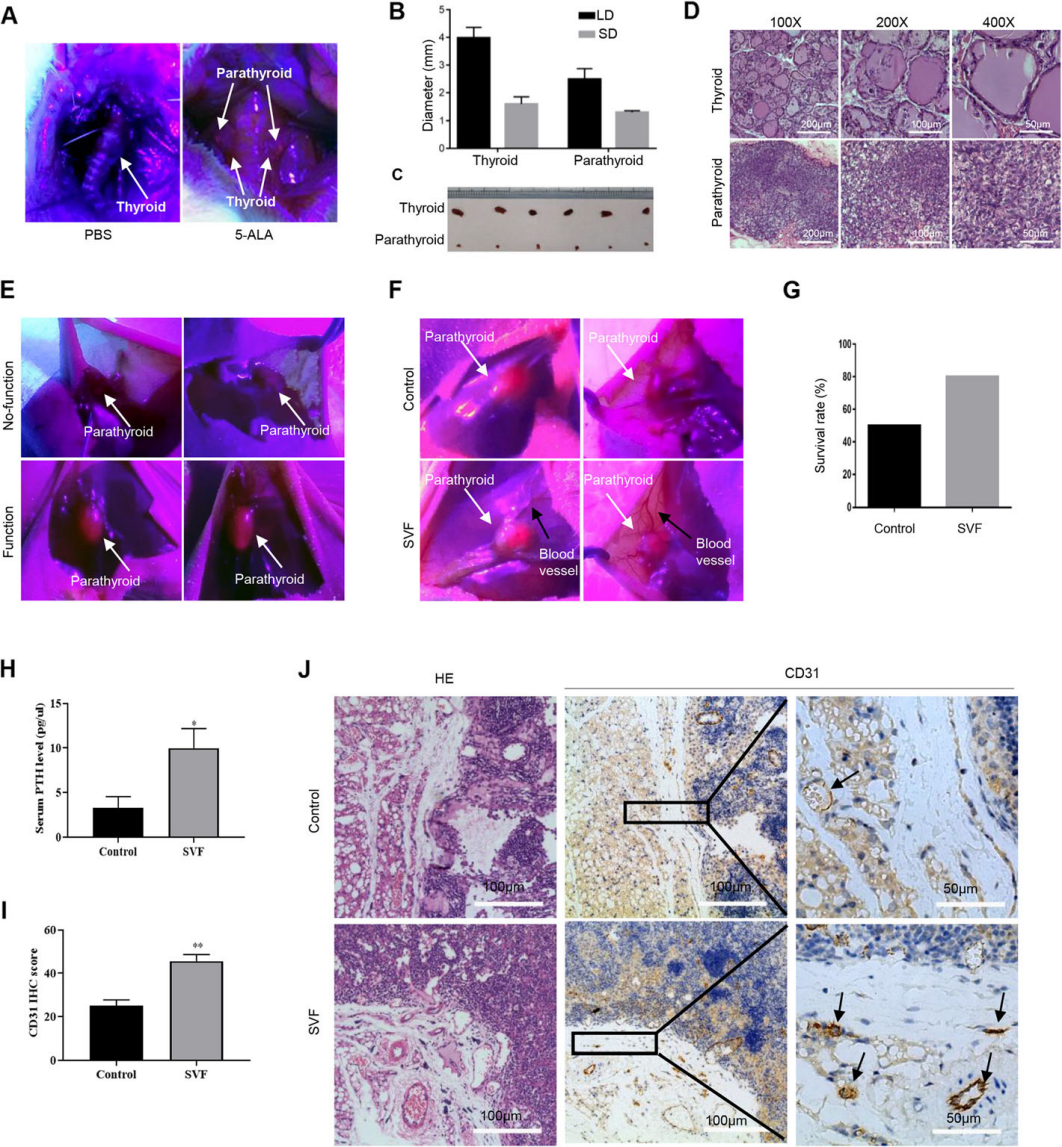
SVFs improved parathyroid transplantation survival and blood vessel assembly. **a** The parathyroid glands were hard to be distinguished from thyroid under normal white light in the rat, while 5-ALA sufficiently visualized the parathyroid gland pairs with red fluorescence under blue light illumination. **b** The long and short diameters of thyroid gland lobe were approximately 4 mm × 2 mm, while those of parathyroid gland was approximately 2 mm × 1 mm. LD, long diameter; SD, short diameter. **c** The thyroid glands were brownish red, and the parathyroid glands were smaller and lighter color. **d** Pathological examination confirmed the efficacy of 5-ALA methods to identify parathyroid. **e** 5-ALA was used to visualize the functional transplanted parathyroid grafts, which exhibited red fluorescence under blue light. **f** More blood vessels around the transplanted parathyroid tissues in the SVF group. **g** The survival rate of the SVF group was higher than that of the control group. **h** The serum PTH levels in the SVF group was 2-fold higher than those in the control group, *n* ≥ 5; *p < 0.05. **i** Quantification of vessel analysis throughCD31 IHC score calculation of parathyroid grafts in the SVF and control groups, *n* ≥ 5; **p < 0.01. **j** Representative hematoxylin and eosin staining and CD31 IHC staining of parathyroid grafts from the SVF and control groups

Five-aminolevulinic acid (5-ALA) is a precursor of fluorescent and phototoxic protoporphyrin IX (PpIX) in the heme biosynthesis pathway. Due to the optical properties of PpIX and the sensitivity of its synthesis to the intracellular metabolic activity, it has potential to improve the identification of parathyroid gland tissue intraoperatively and no phototoxicity during drug distribution because of its fast pharmacokinetics. The parathyroid glands were hard to be distinguished from the thyroid under normal white light in the rats, while they were visualized with red fluorescence under a blue light (380–440 nm) source after 5-ALA treatment (Fig. 1a). Rats have one pair of parathyroid glands, each of which is approximately 1–2 mm in diameter, much smaller than those of the thyroid glands (Fig. 1b, c). The isolated parathyroid tissues were confirmed with hematoxylin and eosin (HE) staining by experienced pathologists (Fig. 1d). Functional parathyroid grafts were easily indicated by 5-ALA (Fig. 1e). No false positives occurred in the study.

Both parathyroid glands and SVFs were isolated from rats and transplanted into nude mice. All nude mice in the control (*n* = 10) and SVF groups (*n* = 10) were euthanized 8 weeks after transplantation of rat parathyroid glands. The survived transplanted grafts were easily identified with red fluorescent via 5-ALA photosensitization (Fig. 1f). Five of 10 mice (50%) in the control group and 8 of 10 mice (80%) in the SVF group were detectable of the red fluorescence, which indicated higher success rate for parathyroid xenotransplantation and functional parathyroid grafts with SVFs co-transplantation (Fig. 1g). The serum levels of PTH were indeed examined to assess transplanted graft function. The serum levels of rat PTH in the SVF group were significantly higher (12.50 ± 1.78 pg/ml, *n* = 8) than those in the control group (6.62 ± 1.24 pg/ml, *n* = 5, *p* = 0.0368, Fig. 1H). Neovascularization (black arrows) was easily observed around the transplanted parathyroid tissues (white arrows) in the SVF group (Fig. 1f). The vessel profiles were compared between the SVF and control groups from similar positions in the transplanted grafts. There was a significant increase in blood vessels indicated by CD31 staining around the transplanted parathyroid tissues in the SVF group (45.63 ± 3.07 per area, *n* = 8) compared with the control group (25.00 ± 2.28 per area, *n* = 5, *p* < 0.01, Fig. 1i, j).

### SVFs induce angiogenic factors FGF2 and VEGF-A in the parathyroid grafts

**Fig. 2:**
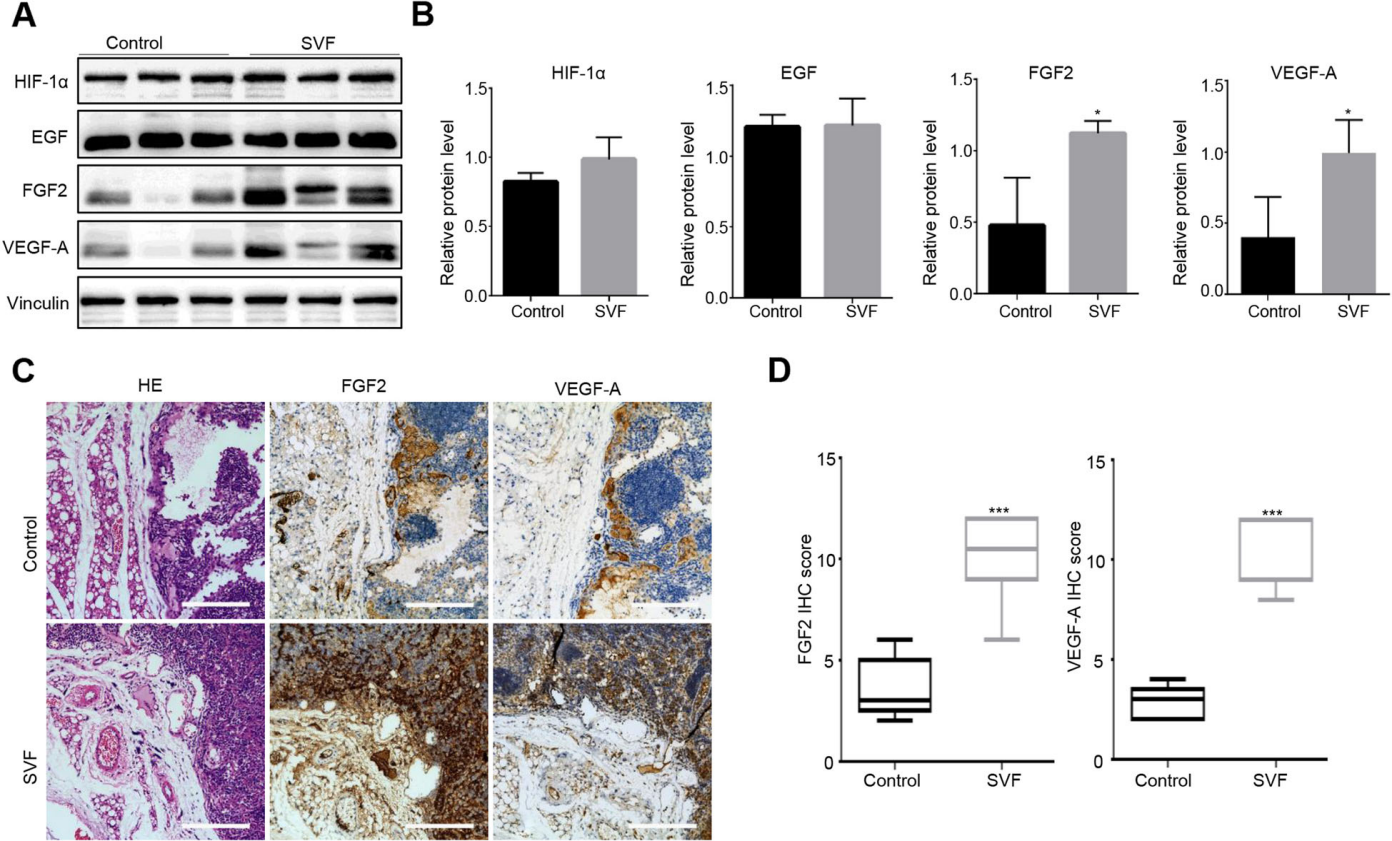
SVFs induced FGF2 and VEGF-A expression levels in the parathyroid grafts. **a** Immunoblotting of HIF-1α, EGF, FGF2, VEGF-A, and vinculin in the parathyroid tissues from both the control and SVF groups. **b** Quantification of the protein levels of HIF-1α, EGF., VEGF-A (*p* = 0.019), and FGF2 (*p* = 0.034), *n* = 3; *p < 0.05. **c** Representative IHC images of the parathyroid grafts from both the SVF and control groups. Scale bar, 100 μm. **d** Higher FGF2 and VEGF-A IHC scores in the SVF group compared with the control group, *n* ≥ 5; ***p < 0.001

To further explore the mechanism of SVF-induced angiogenesis, we used immunohistochemical (IHC) staining and immunoblotting to examine several angiogenic factors in the transplanted parathyroid tissues. HIF-1α and EGF were detected, but no differences were found between the control and SVF groups (*p* = 0.640 and 0.571, respectively, Fig. 2a, b). The SVF group showed significantly increased FGF2 (*p* = 0.034) and VEGF-A expression (*p* = 0.019) compared with the control group (Fig. 2a, b). IHC staining confirmed that both FGF2 and VEGF-A levels were higher compared with the control group (*p* < 0.001, *n* ≥ 5, Fig. 2c, d).

### Higher microvessel density and increased VEGF-A levels are associated with the graft survival of human parathyroid tissues

**Fig. 3:**
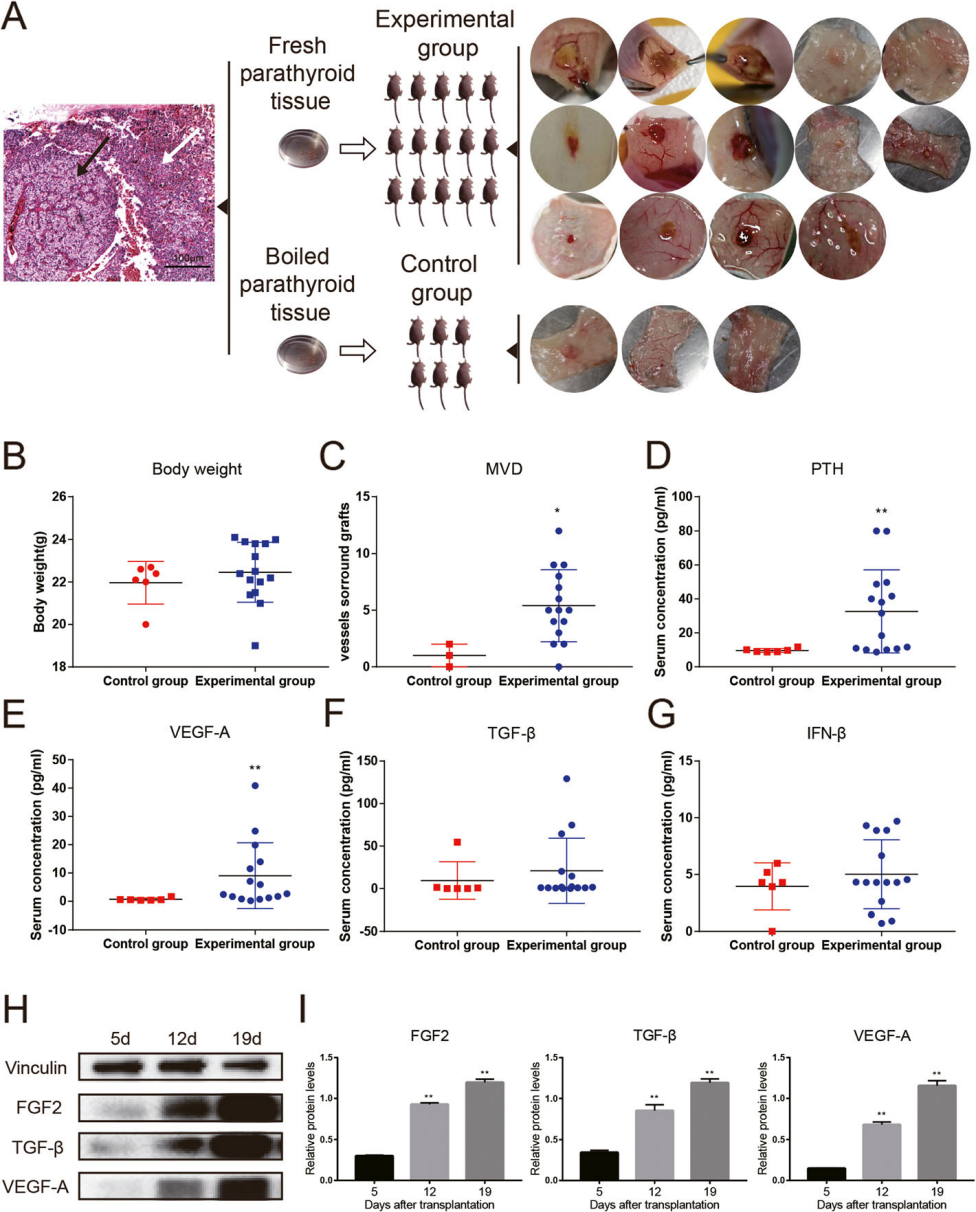
Functional human parathyroid grafts had higher microvessel density and increased VEGF-A expression. **a** Human parathyroid tissues identified by H&E staining (write arrow, normal tissue; black arrow, abnormal tissue), and the normal tissues were transplanted into nude mice: 15 mice with fresh tissues and 6 mice with boiled tissues, resulting in 14 samples found in the fresh group and 3 samples in the boiled group. **b** The body weight of nude mice was comparable between the fresh and boiled groups. **c** Microvessel density (MVD) around parathyroid grafts was significantly higher in the fresh group compared with the boiled group (*p* = 0.0337). **d**–**g**. Serum levels of PTH (**d**, *p* = 0.0063), VEGF-A (**e**, *p* = 0.0048), TGF-β (**f**), and INF-β (**g**) were measured by ELISA. *n* ≥ 3; *p < 0.05, **p < 0.01. **h** Representative immunoblotting of vinculin, FGF2, TGF-β, and VEGF-A in the functional parathyroid grafts 5, 12, and 19 days after transplantation. **i** Quantification of relative protein levels of FGF2, TGF-β, and VEGF-A. TGF-β and VEGF-A were upregulated after transplantation, *n* = 3; **p < 0.01

To confirm the crucial roles of vessel assembly and angiogenesis during parathyroid transplantation, human parathyroid tissues were transplanted into nude mice and microvessel density and angiogenic markers were examined. Fifteen mice were transplanted with fresh human parathyroid tissues and 6 mice with boiled ones. There were 17 grafted tissues finally found while others were absorbed (Fig. 3a). No animal in both groups died within 19 days after transplantation. The mean weight of male nude mice was 22.46 ± 0.36 g in the experimental group and 21.96 ± 0.50 g in the control group (*p* = 0.48, Fig. 3b). Microvessel density was greater in the experimental group than the control group (*p* = 0.045, Fig. 3c). The serum levels of human PTH significantly raised (*p* = 0.044) in the experimental group (Fig. 3d). Similarly, the serum levels of VEGF-A in the experimental group were significantly higher than those in the control group (Fig. 3e), while no significant difference for TGF-β or IFN-β (Fig. 3f, g). However, all the levels of FGF2, TGF-β, and VEGF-A significantly raised over time after transplantation (Fig. 3h, i).

### Hypoxia induced angiogenic factors in ADSCs from SVFs via EYA1

**Fig. 4:**
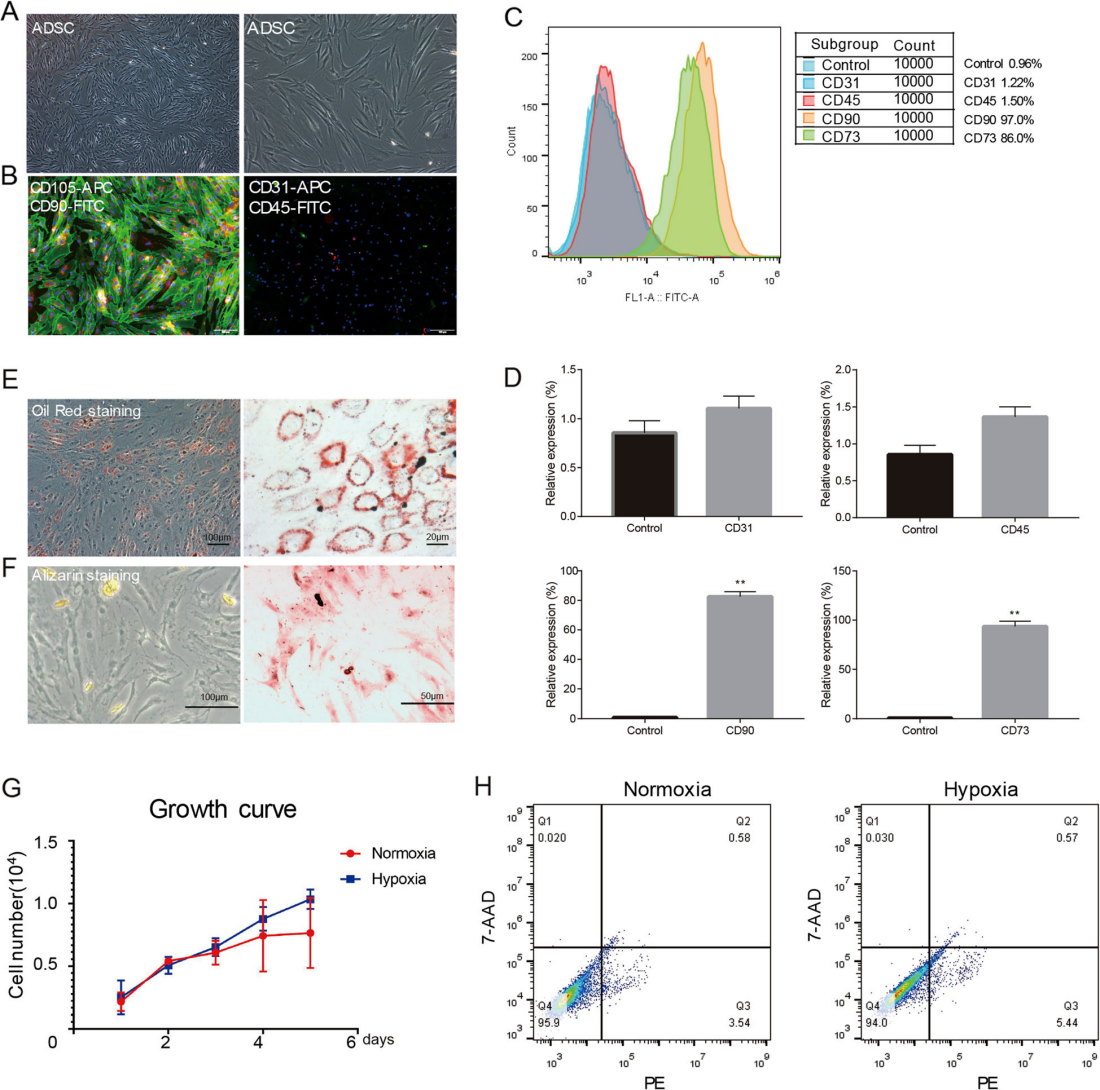
Hypoxia had no effect on proliferation and apoptosis of human adipose-derived stromal cells. **a** ADSCs had fibroblast-like and spindle-like morphology. **b** Representative immunofluorescent staining of CD31, CD45, CD90, and CD105. Scale bar, 100 μm. **c** Representative flow cytometry of CD31 (1.22%), CD45 (1.50%), CD90 (97.0%), and CD73 (86.0%) in ADSCs. IgG was used as the isotype control. **d** Quantification of cytometry showed that ADSCs were CD31−/CD45−/CD73+/CD90+, *n* ≥ 3; **p < 0.01. **e** Representative Oil Red O staining of adipogenesis. **f** Representative Alizarin Red S staining of osteogenesis. **g** Cell Counting Kit-8 assay suggested no significant difference in ADSC proliferation, *n* = 3, *p* > 0.05. **h** Representative flow cytometric analysis of Annexin V suggested no significant difference in ADSC apoptosis

**Fig. 5:**
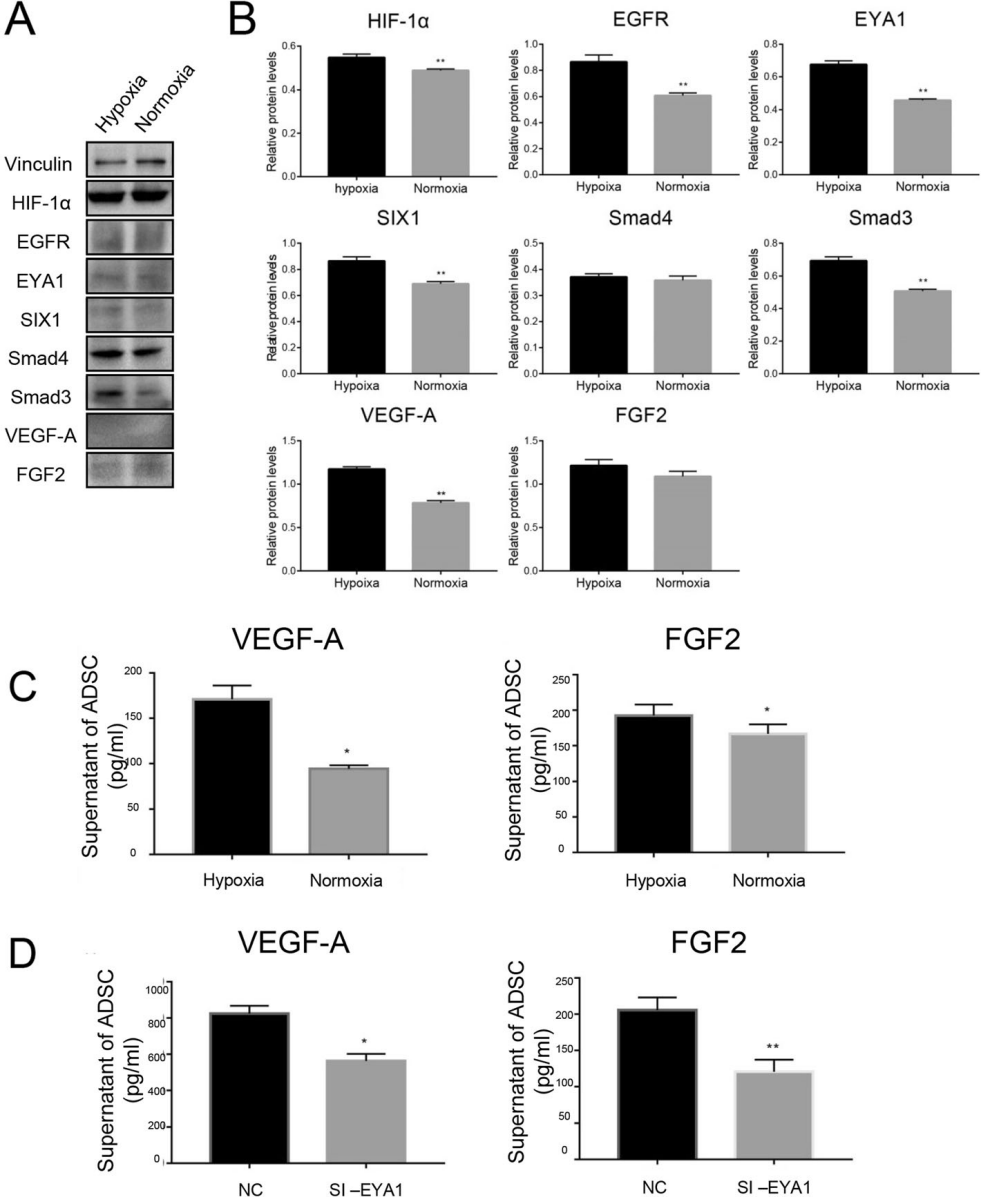
EYA1 modulated hypoxia-induced angiogenic factor production and secretion in ADSCs. **a** Representative immunoblotting of ADSCs cultured in either hypoxic or normoxic condition. **b** Quantification of immunoblotting, *n* = 3; **p < 0.01. **c** ELISA of VEGF-A and FGF2 in ADSCs cultured in either hypoxic or normoxic condition, *n* = 3; *p < 0.05. **d** Knockdown of EYA1 decreased VEGF-A and FGF2 levels in the supernatant of ADSCs, *n* = 3; *p < 0.05; **p < 0.01

SVFs are composed of mixed cell populations including vascular endothelial cells, adipose progenitor cells, stromal cells, and blood cells. To confirm that ADSCs existing in the SVFs are involved in the induced angiogenesis of parathyroid grafts co-transplanted with SVFs, human ADSCs were isolated from human SVFs as previous reports. The morphology of isolated ADSCs was fibroblast-like and spindle-like shape (Fig. 4a). Immunofluorescence confirmed that they are CD90 and CD105 positive, while CD31 and CD45 negative (Fig. 4b–d). The isolated ADSCs had the potential to differentiate into adipocytes and osteoblasts (Fig. 4e, f). The transplanted tissues often suffer from low oxygen supply due to insufficient blood vessels. Therefore, we investigated the effects of hypoxia on ADSCs. Proliferation and apoptosis analyses indicated no effect of hypoxia on ADSC division and programed death (Fig. 4g, h). However, the lack of oxygen induced angiogenic factors in the ADSCs, including HIF-1α and VEGF-A (Fig. 5a, b). Interestingly, upregulation of EYA1 and SIX1 was also observed, suggesting DACH1/SIX1/EYA1 pathway might be involved in the modulation of angiogenesis triggered by hypoxia in ADSCs. ELISA results indicated more VEGF-A and FGF2 levels in the supernatant of hypoxic ADSCs (Fig. 5c). Moreover, knockdown of EYA1 suppressed the production and secretion of these angiogenic factors in ADSCs (Fig. 5d).

### The supernatant of ADSCs induced tubule formation and migration of human endothelial cells

**Fig. 6:**
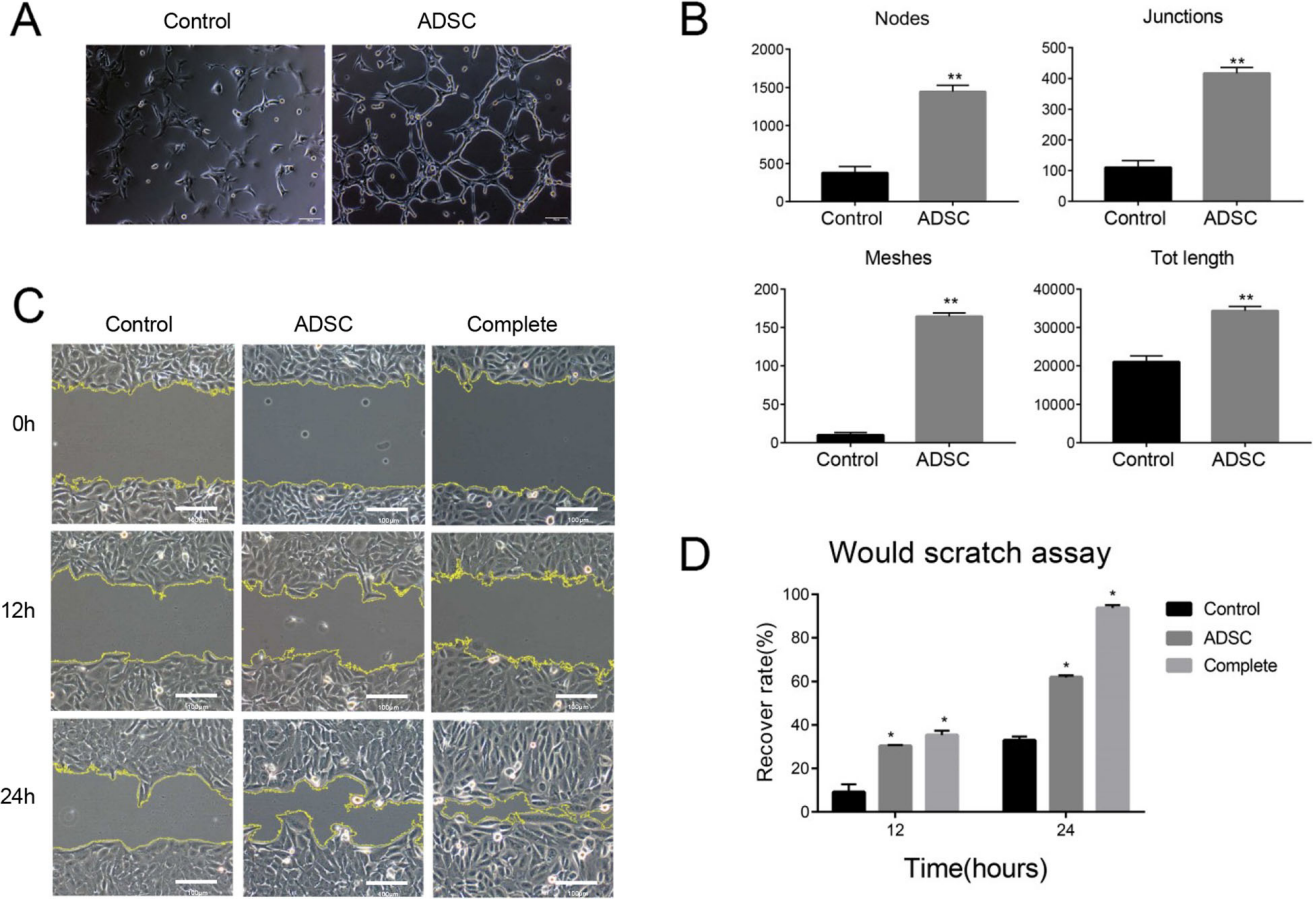
The supernatant of ADSCs induced tubule formation and migration of HUVECs. **a** Representative images of HUVEC tubule formation om Matrigel. Scale bar, 100 μm. **b** Quantification of node numbers, junction numbers, mesh numbers and total length of tubule structures, *n* = 3; **p < 0.01. **c** Representative images of scratch wound healing assay. Scale bar, 100 μm. **d** Quantification of wound scratch assay, *n* = 3; *p < 0.05

To further test the functional roles of ADSCs during angiogenesis, the effects of ADSC supernatant on human umbilical vein endothelial cell (HUVEC) behaviors were examined. The results indicated that the supernatant of ADSCs increased HUVEC tubule formation (Fig. 6a, b). Endothelial cell migration is closely involved in the process of angiogenesis. Wound healing assay showed that the supernatant of ADSCs improved HUVEC migration (Fig. 6c, d). Our results suggested that the angiogenic factors secreted by ADSCs induced human endothelial cell functions.

### ADSC induced angiogenesis via EYA1 in mice model of parathyroid transplantation

**Fig. 7:**
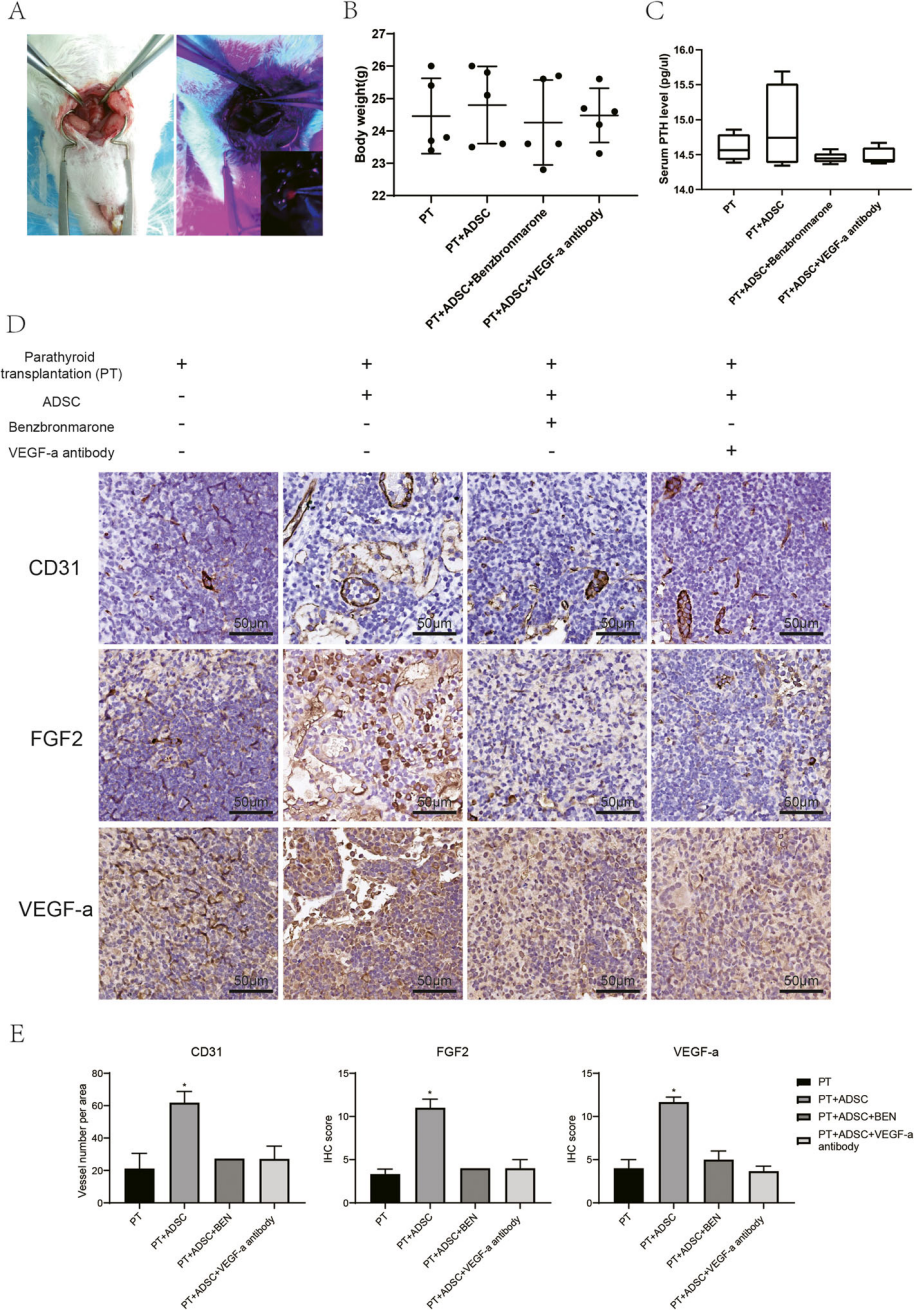
ADSCs promoted the survival and angiogenesis of parathyroid grafts via inducing FGF2 and VEGF-A through EYA1. **a** The parathyroid glands were removed from rats and transplanted into nude mice. **b** The body weights of mice were comparable. **c** The serum levels of PTH were measured with rat PTH ELISA kit. ADSCs induced PTH levels, and EYA1 inhibitor and VEGF-A antibody abolished this induction, *n* ≥ 4. **d** Representative immunochemistry staining of CD31, FGF2, and VEGF-A in parathyroid grafts. **e** Quantification of vessel numbers per area, as well as FGF2 (surface) and VEGF-a (cytoplasm) levels, *n* ≥ 3, *p < 0.05

There were 24 pairs of rat parathyroid glands transplanted in 24 nude mice. They were divided into 4 groups, 5 mice of merely parathyroid transplantation, 6 mice of co-transplantation with ADSC, 6 mice of co-transplantation with benzbromarone (MedChemExpress, HY-B1135, China; EYA1 inhibitor) treated ADSC, and the other 7 mice of co-transplantation with VEGF-A antibody-treated ADSC. All nude mice were euthanized 8 weeks later after transplantation; 3 mice in parathyroid only group were found with parathyroid tissue, 4 mice in ADSC co-transplantation group, 3 mice in benzbromarone-treated group, and 2 in VEGF-A antibody-treated group. The CD31, VEGF-A and FGF2 staining showed that the ADSC group had a higher CD31, VEGF-A, and FGF2 level than the other two groups. But the serum PTH values did not show much difference between these four groups (Fig. 7).

## Discussion

Parathyroid transplantation was developed to cure permanent hypoparathyroidism after parathyroid surgery to avoid severe hypocalcemia and to reduce or stop supplementation treatment. The efficacy of early autotransplantation in primary hyperparathyroidism was estimated at 85–99% [36].. Previous studies revealed a wide variation in the proportion of functional grafts. A retrospective study indicated that the median duration of storage was 11.1 months (range, 0.4–28.5), and 80% were nonfunctional at 26 months median follow-up [37]. Furthermore, both in vitro and in vivo studies showed that very few parathyroid samples are potentially functional after 2 years of cryopreservation [38, 39]. These researches suggested the survival rate of transplantation should be effectively improved.

Several methods have been used to promote transplanted tissue angiogenesis. Yazawa et al. reported that free-fat graft survival could be improved by adding basic fibroblast growth factor delivered by a cylinder-type silicone substrate [40]. Ding et al. demonstrated that microspheres loaded with VEGF significantly promoted fat graft neovascularization, thus improving adipocyte survival [41]. Park et al. applied small intestinal submucosa to PA in a rat model of hypoparathyroidism to promote angiogenesis surrounding transplanted parathyroid tissues [42]. The applications of SVFs have been relatively mature in the field of esthetic plastic surgery, but not in the field of organ transplantation. Our results suggested that the survival rate of transplanted parathyroid with SVFs was 30% higher than the control. The higher survival rate of the SVF group might result from the increased early blood supply. Our IHC and immunofluorescence experiments demonstrated that SVFs markedly induced angiogenesis around the transplanted grafts and that SVFs improved vascular assembly of the transplanted parathyroid glands via increasing FGF2 and VEGF-A expression in vivo. Therefore, supplementation of adipose-derived SVFs provides a feasible strategy in parathyroid transplantation by promoting neovascularization around transplanted parathyroid tissues.

The apoptosis and proliferation of hypoxia-conditioned ADSC seemed not be changed obviously compared to normoxia, which was controversial to some reports [43, 44]. It is also unclear whether the proliferation or differentiation capability was better maintained in lower oxygen concentration [19]. In our study, the proliferation of hypoxia-conditional ADSCs was not improved and the apoptosis not reduced with significant difference compared to normoxia. ADSCs naturally reside in specific niches with low oxygen tension, less than 4% O_2_ present in human adipose tissue [45]. The oxygen tension was supposed to be an important role in ADSC cultivation in vitro. In addition, ADSCs undergo more populations doubling than mesenchymal stem cells [46], expressing diverse membrane markers and offering diverse advantages for repairmen. They are not restricted to differentiation into chondrocytes and osteocytes in vitro [47]. So, the conditions of O_2_ tension should be controlled strictly while investigating specific potentials of ADSCs in further studies.

EYA family are involved in many cellular processes, including both cell proliferation, migration, and invasion [25]. Our recently published research showed that EYA1 is necessary for STAT3 signal activation and SIX1-induced cell proliferation. It is suggested that SIX1 may not directly activate gene transcription but there is mutual regulation between SIX1 and the EYA family instead [48]. HIF-1α is one of the most important upstream genes of VEGF-A [28]. EYA1 regulates VEGF-A through upregulating HIF-1α at the transcription level. HIF-1α and HIF-1β induce the transcription of more than 70 downstream target genes during tumorigenesis, but EYA1 cannot regulate HIF-1β expression [31]. EYA1 is a co-transcription activator with intrinsic protein phosphatase activity and binds DNA through a binding medium, such as SIX [49]. One of the most important regulators of HIF-1α is The PI3K/AKT pathway [50]. Since phosphorylation of EYA1 by PI3K/Akt signaling enhances EYA1 transcription activity, EYA1 may enhance angiogenesis through the PI3K/AKT pathway.

EYA1 was reported to regulate VEGF-A during angiogenesis through HIF-1α signaling pathway in a cancer model, which is consistent with our findings in the PA animal model. ADSCs induced PA survival and vessel assembly, while EYA1 inhibitor and VEGF-A antibody abolished this induction. These results suggested that EYA1 regulated PA angiogenesis through VEGF-A.

With multipotent differentiation potent and angiogenic ability, ADSC is also quite convenient and easily available; our research showed that ADSC is promising in parathyroid transplantation application. In addition, EYA1 expression level is not only related to VEGF-A expression, and it is also a key regulator in parathyroid development [25]. In other words, because EYA1 is necessary for parathyroid development and played a role in the angiogenic procedure, if ADSC could be induced differentiating into parathyroid cells, it could be a perfect resource for clinical employment in the treatment of hypoparathyroidism. It could also provide a potential and specific target to improve transplantation and differentiation in other tissues.

## Conclusions

In the present study, we reported that ADSCs from adipose-derived SVFs improved the survival of parathyroid transplantation and vascular assembly parathyroid grafts. EYA1 might play a role in angiogenesis by coordinating with HIF-1α and regulated by PI3K/AKT pathway, which needs further detection. Our results suggested the therapeutically potential application of ADSCs and SVFs during the parathyroid transplantation.

## Data Availability

All data generated or analyzed during this study are included in its supplementary information files.

## Abbreviations

SVFs: Stromal vascular fractions
ADSCs: Adipose-derived stem cells
VEGF: Vascular endothelial growth factor
FGF: Fibroblast growth factor
EYA1: The eyes absent homolog 1
PTH: Parathyroid hormone
PA: Parathyroid autotransplantation
HIF: Hypoxia-inducible factor
MSC: Mesenchymal stem cell
ELISA: Enzyme-linked immunosorbent assays
SD: Sprague-Dawley
PBS: Phosphate buffered saline
DMEM: Dulbecco’s modified Eagle’s medium
CCK-8: Cell Counting Kit-8 assay
5-ALA: five-aminolevulinic acid
PpIX: Phototoxic protoporphyrin IX
HE: Hematoxylin and eosin
IHC: Immunohistochemical
HUVECs: Human umbilical vein endothelial cells
